# Chronic aortic dissection with tricuspid regurgitation: report of a case

**DOI:** 10.1002/ccr3.1343

**Published:** 2017-12-22

**Authors:** Ippei Takazawa, Shin‐ichi Ohki, Yoshio Misawa

**Affiliations:** ^1^ Division of Cardiovascular Surgery Department of Surgery Jichi Medical University Shimotsuke Tochigi Japan

**Keywords:** Annuloaortic ectasia, aortic dissection, tricuspid annuloplasty, tricuspid regurgitation

## Abstract

Dilatation of aortic root may distort the tricuspid annulus. We experienced a case of chronic aortic dissection presented with tricuspid regurgitation. Chest computed tomography revealed an enlarged ascending aorta displacing the right heart. The patient successfully underwent replacement of the aorta and tricuspid annuloplasty with a prosthetic annulus.

## Introduction

Secondary tricuspid valve disease commonly occurs mainly in combination with left‐sided heart valve disease. Volume overloading and/or pressure overloading of the right ventricle can cause the regurgitation. A left‐to‐right shunt at the atrium can also cause it. Proximal extension of type A aortic dissection can affect the aortic valve and affects the tricuspid valve functions. A case of chronic aortic dissection presented with tricuspid valve regurgitation (TR). Preoperative chest computed tomographic findings revealed a tortuous change of the tricuspid annulus. We assess tricuspid annulus morphology and tricuspid valve function.

## Case Presentation

**Figure 1:**
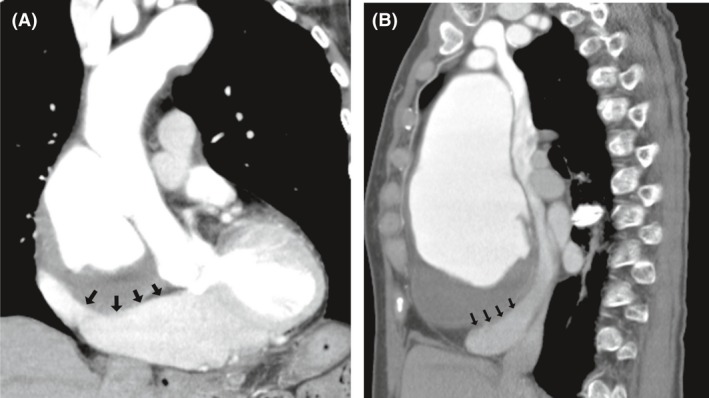
Preoperative contrast‐enhanced chest computed tomography. A (frontal view) and B (sagittal view) shows that a dilatation of the ascending aorta with dissection is observed. The right heart is stretched and distorted by the dilated false lumen (arrows).

A 74‐year‐old woman was admitted for treatment of a type A chronic aortic dissection. The patient suffered with chest discomfort for several months, and she saw her family physician because of the persistent discomfort. A physical examination on admission showed no remarkable findings. A blood test showed normal renal and liver functions without anemia. Chest X‐ray showed an enlarged cardiothoracic ratio of 75% with clear lung fields. Electrocardiography showed normal sinus rhythm without left ventricular hypertrophy. A preoperative echocardiography showed moderate TR with dilatation of the sinus of Valsalva. The aortic valve and left ventricle functioned well. Pericardia effusion was slightly observed around the right ventricle. Chest computed tomography revealed an enlarged ascending aorta of 73 × 71 mm in diameter distorting and stretching the right heart (Fig. 1A and B). The aortic arch was also involved. Pleural and pericardial effusion was not observed.

The patient gave an informed consent for surgical treatment. She underwent replacement of the ascending aorta and aortic arch under cardiopulmonary bypass through a standard full sternotomy. The dilated tricuspid annulus was also repaired with a prosthetic annulus. Fibrous pericardial adhesion around the ascending aorta might endorse chronic features of the dissection.

**Figure 2:**
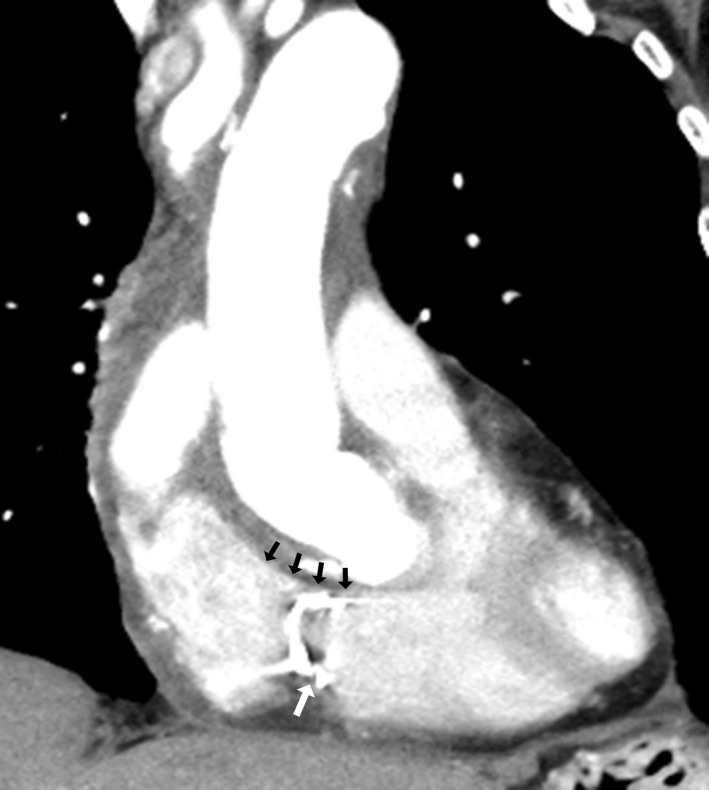
Postoperative contrast‐enhanced chest computed tomography. The right heart returns to a normal contour (black arrows), and the tricuspid annulus is repaired with a prosthetic annulus (white arrow).

Postoperative chest computed tomography showed the normalized right heart and the tricuspid annulus with a prosthetic annulus (Fig. 2). Echocardiography revealed the TR disappeared. The patient's postoperative course was uneventful, and she was doing well 3 months after the operation.

## Discussion

Tricuspid valve annuloplasty is the treatment of choice for TR secondary to left‐sided heart valve diseases, and its excellent clinical results provide cardiac surgeons an active posture for repair of TR in case of a left heart surgery 1, 2, 3, 4, 5. In such a case, annular dilatation of the tricuspid valve contributes to TR. Residual TR affects the long‐term prognosis after left‐side valve surgeries. Today, tricuspid annulus repair with a prosthetic ring is a standard procedure for a case with both a left‐sided heart valve disease and TR.

Aortic diseases can also lead to TR. Dilatation of aortic root may distort the tricuspid annulus. Some investigators reported acute aortic dissection cases with TR 6, 7. Kurisu and colleagues concluded that a tricuspid annulus distortion might cause TR in their case 6. After grating of the ascending aorta in an acute case, the distorted tricuspid annulus might return to a normal form. Our case was chronic, and the annulus was dilated. Then, we repaired the annulus with a prosthetic partial ring.

Other aortic root diseases include aneurysms. Bulkley and colleagues reported a case of aneurysm of the sinus of Valsalva causing tricuspid valvular dysfunction 8. They inferred the aneurysm as a cause of obscure right‐sided valvular disease. Akashi and colleagues showed a Marfan patient with annuloaortic ectasia, mitral regurgitation, and tricuspid regurgitation in a patient 9. They did not discuss the relation between the causes of TR and the aortic dilatation. But, a dilated sinus of Valsalva had a potential risk for TR in their case.

Identifying a cause of TR in a case with an aortic root enlargement requires evidences of a tortuous tricuspid annulus on echocardiographic and/or radiographic examinations. We inferred that the dilated aortic root with the coagulated false lumen made the tricuspid annulus deformed, causing tricuspid regurgitation. We could not point out the tortuous annulus on echocardiography, but computed tomographic findings revealed a deformed right heart, which might affect the tricuspid annulus and valve function. A deformity around the commissure between the anterior and septal cusps could lead to tricuspid valve regurgitation. Morphological features of the tricuspid valve often prevent us from getting its whole valve function in detail. A functional distortion of the tricuspid valve might been reversed by the aortic root surgery. However, the tricuspid annulus was dilated in our case, and we were afraid that the distorted tricuspid annulus would not be reversed postoperatively. Therefore, we repaired the tricuspid annulus. As transcatheter intervention for tricuspid valve regurgitation 10 was not included in our present strategy, we repaired the tricuspid annulus simultaneously with the aortic surgery.

## Conclusions

We experienced a case of chronic aortic dissection presented with TR. We inferred that the enlarged sinus of Valsalva displaced the right heart, causing TR. Grafting of the dilated aorta and TR repair surgery led to excellent results.

## Authorship

IT and SO: participated in the design this study and helped competing an initial manuscript. **Y**M**:** carried out its final manuscript. All authors read and approved the manuscript.

## Conflict of Interest

None declared.
